# Case of Calculi in Perineum and Bladder

**Published:** 1849-03

**Authors:** Owen Munson


ART. IV.—*Case of Calculi in Perineum and Bladder.* Lithotomy by Prof. Eve, of Georgia. Communicated in a letter to Prof. Hamilton, by Owen Munson, M. D.

Augusta, Ga., Jan. 12th, 1849.

Dear Doctor:—As you are devoted to surgery, I have thought you might be interested in the report of a case that I have recently witnessed. I have availed myself of the polite invitation of the Professor of Surgery, and others of the Faculty, to attend the Clinical Lectures, and surgical operations, &c., as my time or inclination might dictate. I was present at the Hospital last Saturday (6th inst.) to witness the operation of lithotomy, by Dr. Paul F. Eve, Professor of Surgery in the Georgia Medical College located in this city. The patient, Mr. Samuel O'Bannon, is a man of about 40 years of age. According to his own story, his back was injured by the falling in of the roof of a building in process of erection some 24 years ago. Partial paralysis of the lumbar region, and consequently, difficult micturation, immediately supervened. But for the last few years urination has been attended with the greatest difficulty, and most excruciating pain.

During the years 1845 and '6 he was enabled to urinate only in the sitting position; and for the last two years he has been compelled to lie down on his right side, place his left foot on his right thigh, introduce the index finger of the left hand into the rectum, and by a sudden motion of the finger throw one or more calculi forward, which would allow a small quantity of urine to flow, and then the passage would again close up. It was only by continuing these manipulations for hours at a time, that he was enabled to relieve the bladder. Severe prolapsus ani was thus produced. The feel produced by placing the hand, or ends of the fingers on the perineum, was very much like that of pebbles tied up in a buckskin bag. The patient said that when he sat down, the cracking and grinding, produced by the friction of the calculi in the perineum reminded him of sitting down on snow.

So great and for so long a time continued had been his sufferings, that he was worn down to a walking skeleton, and life had become intolerable. He was convinced that nothing but death or surgery could relieve him. And so little of hope and encouragement were held out to him by the latter, taking into account his extreme debility, the long standing and extreme difficulty of the case, and the severity of the operation, he seemed not to care in what way his sufferings were ended. Night and day for almost a quarter of a century, he said he had suffered martyrdom. He finally determined to submit to the operation, as affording his only possible chance for relief. After putting him under the full inflnence of chloroform, Dr. Eve commenced the operation in his usually bold, dexterous and efficient manner. In a very short time the Dr. extracted fifty-seven calculi from the perineum, which were firmly impacted in the cellular tissue. This done, he entered the bladder, from which he extracted sixty more, making a sum total of 117. The largest weighed three drachms, 17 grains. The smallest, less than a pea in size. Aggregate weight, 4℥, 3ʒ, 1℈, and 18 grs. And what is peculiar, every one tetrahedrons, perfectly smooth, and of a dun or leaden color.

Jan. 12.—I have just seen the patient and find him doing well—has walked through a long hall this afternoon, to another room. Wound healing kindly; no offensive smell; tongue clean; appetite good; bowels regular; urinates freely through the urethra; pulse soft and natural,—and in short the poor fellow has every appearance of speedy recovery.

